# Highly Efficient Low-Temperature N-Doped TiO_2_ Catalysts for Visible Light Photocatalytic Applications

**DOI:** 10.3390/ma11040584

**Published:** 2018-04-10

**Authors:** Julien G. Mahy, Vincent Cerfontaine, Dirk Poelman, François Devred, Eric M. Gaigneaux, Benoît Heinrichs, Stéphanie D. Lambert

**Affiliations:** 1 Department of Chemical Engineering—Nanomaterials, Catalysis & Electrochemistry, University of Liège, B6a, Quartier Agora, Allée du six Août 11, 4000 Liège, Belgium; vincent_cerfontaine@hotmail.com (V.C.); b.heinrichs@uliege.be (B.H.); stephanie.lambert@uliege.be (S.D.L.); 2 LumiLab, Department of Solid State Sciences, Ghent University, 9000 Gent, Belgium; Dirk.Poelman@UGent.be; 3 Institute of Condensed Matter and Nanosciences—MOlecules, Solids and ReactiviTy (IMCN/MOST), Université catholique de Louvain, Place Louis Pasteur 1, Box L4.01.09, 1348 Louvain-La-Neuve, Belgium; francois.devred@uclouvain.be (F.D.); eric.gaigneaux@uclouvain.be (E.M.G.)

**Keywords:** N-doped TiO_2_, aqueous sol-gel process, photocatalysis, *p*-nitrophenol degradation, multiple crystalline phase catalyst

## Abstract

In this paper, TiO_2_ prepared with an aqueous sol-gel synthesis by peptization process is doped with nitrogen precursor to extend its activity towards the visible region. Three N-precursors are used: urea, ethylenediamine and triethylamine. Different molar N/Ti ratios are tested and the synthesis is adapted for each dopant. For urea- and trimethylamine-doped samples, anatase-brookite TiO_2_ nanoparticles of 6–8 nm are formed, with a specific surface area between 200 and 275 m^2^·g^−1^. In ethylenediamine-doped samples, the formation of rutile phase is observed, and TiO_2_ nanoparticles of 6–8 nm with a specific surface area between 185 and 240 m^2^·g^−1^ are obtained. X-ray photoelectron spectroscopy (XPS) and diffuse reflectance measurements show the incorporation of nitrogen in TiO_2_ materials through Ti–O–N bonds allowing light absorption in the visible region. Photocatalytic tests on the remediation of water polluted with *p*-nitrophenol show a marked improvement for all doped catalysts under visible light. The optimum doping, taking into account cost, activity and ease of synthesis, is up-scaled to a volume of 5 L and compared to commercial Degussa P25 material. This up-scaled sample shows similar properties compared to the lab-scale sample, i.e., a photoactivity 4 times higher than commercial P25.

## 1. Introduction

Nowadays, environmental issues are a main problem in our lives, and a lot of research is conducted to reduce them. Pollutants can be very diverse molecules, occurring as aromatic compounds, pesticides, chlorinated compounds, heavy metals or petroleum hydrocarbons [1]. Different methods exist to reduce them, such as photocatalysis, which is an efficient process for degrading organic pollution [2].

This technique consists of redox reactions between organic pollutants and radical species produced by the illumination of a photocatalyst [3,4].

The most commonly used photocatalyst is TiO_2_ [1,5], which is a semiconductor sensitive to UV radiation; it is non-toxic and cheap [6]. The photon energy required to activate the TiO_2_ is 3.2 eV (band gap width); this value corresponds to radiation with a wavelength lower than 388 nm (for anatase phase). However, in the case of illumination by solar light, only 5–8% of the spectrum will be used for activation (only the UV part) [7]. When energetic light illuminates TiO_2_, electrons (e-) are promoted from the valence band to the conduction band, leading to the formation of positive holes (h+) in the valence band. When these photoactive species reach the surface of the material, they react with water and oxygen to produce radicals, such as superoxide and hydroxyl radicals, and then these radicals are able to degrade adsorbed organic molecules [4,5,8,9].

In recent years, sol-gel synthesis has been used widely for the preparation of TiO_2_ films or powders allowing a control of the nanostructure and surface properties [6,10,11,12,13]. This process is a soft chemistry method, using low temperature and low pressure, where titanium precursors sustain two main reactions: hydrolysis and condensation [14]. By adapting the rate of these reactions, a colloidal suspension or a solid gel can be obtained.

Sol-gel TiO_2_ materials can be synthesized via several paths depending on the solvent used, which can include organic solvents or water. Usually, when the sol-gel synthesis is conducted in an organic solvent, it is for the purpose of controlling its reactivity by complexation of titanium alkoxide (Ti-(OR)_4_), where R is an alkyl group (–CH_3_, –C_2_H_5_, …). Only a small amount of water is added to avoid precipitation [10,15,16]. Then the material sustains drying and calcination to remove residual organic molecules and to crystallize amorphous TiO_2_ into anatase or rutile phase [17].

When water is used as solvent, peptizing agents are used to form small crystalline TiO_2_ nanoparticles at low temperature [7,18,19]. With this method, crystalline TiO_2_ materials are synthesized at low temperature without organic solvent. This process is thus well adapted for industrialization [8,9].

Indeed, in previous works [8,9], a global process has been developed to produce pure TiO_2_ films deposited on steel at large scale in order to obtain an easy-to-clean surface [8] and doped TiO_2_ with enhanced photocatalytic properties [9]. In these works, pure and doped TiO_2_ aqueous syntheses, using titanium tetraisopropoxide in acidified water, were simplified from [19] to facilitate extrapolation towards an industrial scale, and then pure and doped TiO_2_ sols were scaled up to volumes of 5 L [8,9].

As mentioned above, TiO_2_ needs energetic light to be photoactive, and so, when only the solar spectrum is available for an application, pure TiO_2_ is not very effective. In order to improve the photocatalytic properties of TiO_2_ in the visible range, three main routes can be followed: (i) the introduction of metallic nanoparticles [20] or ions [10,21,22], (ii) the introduction of non-metallic elements like N [23,24,25,26,27,28], B [25,29], F [24,25], P [30,31] or S [24,27], and (iii) doping with dye photosensitizers such as porphyrins [32,33]. 

With regard to the introduction of non-metallic elements, N-doping is considered to be an ideal candidate, because N 2*p* states could effectively mix with O 2*p* states [34]. Indeed, nitrogen can be easily introduced into the TiO_2_ structure, due to its atomic size, which is comparable with that of oxygen, its low ionization energy and high stability [24]. The visible absorption of the N-doped samples is due to the reduction of the band gap. Indeed, substitutional N atoms in TiO_2_ lattice narrow the band gap by creating an energy level above the valence band maximum or creating an intermediate band for the electron below the conduction band [24,26,34].

The aim of the present work is to N-dope the previously developed synthesis [8,9]. This synthesis makes it possible to produce a crystalline TiO_2_ colloid at low temperature in aqueous media. This material can be produced at large scale, showing a high photocatalytic efficiency, but only under UV radiation. Synthesis at pilot scale for N-doped TiO_2_ catalyst by sol-gel process is not reported in the literature. In this work, three N-precursors are used to extend the photoactivity towards visible light: urea, ethylenediamine and triethylamine. The resulting materials are characterized by X-ray diffraction (XRD), transmission electron microscopy (TEM), nitrogen adsorption–desorption measurements, XPS and diffuse reflectance spectroscopy measurements in order to study the crystalline phase formation, the doping influence and the texture of the catalysts. In the second part of this study, the photocatalytic activity of the samples is tested in the degradation of *p*-nitrophenol (PNP, C_6_H_5_NO_3_) in polluted water under visible light to show the influence of N-doping on the photoactivity and identify the best N-precursor for low-temperature N-doping of TiO_2_. For large-scale application, a large volume (5 L) synthesis of the best N-doped TiO_2_ is performed and compared to laboratory-scale synthesis. Although TiO_2_ N-doping has been widely studied, the novelty of this research is, from an easy and environmentally friendly synthesis of photoactive TiO_2_ colloid, to try to N-dope this material in an easy way without heating and to try to up-scale this synthesis to a larger volume for further visible applications. A comparison with commercial Degussa P25 will be made.

## 2. Materials and Methods

### 2.1. Samples Preparation

#### 2.1.1. Pure TiO_2_ Powder Synthesis

Titanium (IV) tetraisopropoxide (TTIP > 97%, Sigma-Aldrich, Saint Louis, MO, USA), nitric acid (HNO_3_, 65%, Merck, Kenneth Fort Worth, NJ, USA), isopropanol (IsoP, 99.5%, Acros, Hull, Belgium) and distilled water are used as starting materials. Pure TiO_2_ is synthesized according to Mahy et al. [8] using water as solvent and nitric acid for TTIP peptization. After a reaction time of 4 h, a light blue transparent liquid sol is obtained. Then, the sol is dried under an ambient air flow to obtain a xerogel, which is crushed in a white-yellow powder [8]. Pure TiO_2_ sample is denoted as “TiO_2_ pure A”. 

An alternative synthesis of pure TiO_2_ is also tested. In this case, a mixture of TTIP and isopropanol is precipitated in water. Then this precipitate is washed three times with water. This slurry is then mixed in water with HNO_3_ (pH = 1) at 50 °C for 12 h. After this reaction time, a light blue transparent liquid sol is obtained. Then, the sol is dried under an ambient air flow to obtain a xerogel, which is crushed in a white-yellow powder [8]. This pure TiO_2_ sample is denoted as “TiO_2_ pure B”. 

#### 2.1.2. N-Doped TiO_2_ Powder Synthesis

TiO_2_ catalyst is doped by nitrogen with three different precursors: urea ((NH_2_)_2_CO, Sigma-Aldrich, 98%), ethylenediamine (NH_2_CH_2_CH_2_NH_2_, Sigma-Aldrich, puriss. p.a., absolute, ≥99.5% (GC)), and triethylamine ((C_2_H_5_)_3_N, Sigma-Aldrich, ≥99%). 

For urea doping, distilled water is mixed with urea then acidified by HNO_3_ to a pH equal to 1. Then, TTIP is added to IsoP, and the mixture is stirred at room temperature for 30 min. The TTIP/IsoP mixture is added to acidified urea/water solution under vigorous stirring. The solution stays under stirring for 4 h at 80 °C. After this time, a light white-blue transparent liquid sol is obtained and kept in ambient atmosphere. The molar ratio between urea and TTIP is equal to either 1, 2, 4 or 10 leading to four urea-doped samples. The samples are called “TiO_2_/UX”, with X corresponding to the molar ratio between urea and TTIP.

For ethylenediamine doping, TTIP/IsoP solution is precipitated in water containing ethylenediamine. The precipitate is washed three times with water. Then it is mixed in water with HNO_3_ (pH = 1) at 50 °C for 12 h. After reaction, a light blue transparent liquid sol is obtained. The molar ratio between ethylenediamine and TTIP is equal to either 1, 2, 4 or 10 leading to four ethylenediamine-doped samples. The samples are called “TiO_2_/EtDNX”, with X corresponding to the molar ratio between ethylenediamine and TTIP.

For triethylamine doping, a pure TiO_2_ sol is synthesized like TiO_2_ pure B sample. Then this colloid is mixed with an excess of triethylamine for 12 h. A yellowish opaque liquid sol is obtained. The molar ratio between triethylamine and TTIP is equal to 28 or 42, leading to two trimethylamine-doped samples. The samples are called “TiO_2_/Et_3_NX”, with X corresponding to the molar ratio between triethylamine and TTIP.

All N-doped samples are dried under ambient air at room temperature, then dried at 100 °C for 1 h. The resulting powders are washed three times with water, then finally dried at 100 °C for 12 h.

#### 2.1.3. Urea-Doped TiO_2_ Powder Synthesis at a Large Scale

The synthesis of the TiO_2_/U2 sample is scaled up to a volume of 5 L in a glass batch reactor with a water recirculation cooling system (jacketed reactor) [9]. 3.6 L of a solution urea/distilled water is acidified with HNO_3_ to a pH equal to 1. Then, 480 g of TTIP is added to 168.8 g of isopropanol (IsoP). The mixture is stirred at room temperature for 30 min. The TTIP-IsoP mixture is added to the acidified urea water and stirred by a propeller at 300 rpm. The liquid stays under stirring for 4 h at a temperature of 80 °C. After the reaction, a light white-blue transparent liquid sol is obtained, similar to the laboratory-scale sol. As for the laboratory-scale TiO_2_ sample, the large-scale urea-doped TiO_2_ catalyst is dried under ambient air at room temperature, then dried at 100 °C for 1 h. The resulting powder is washed three times with water, then finally dried at 100 °C for 12 h.

The large-scale urea-doped TiO_2_ sample is denoted as “TiO_2_/U2-LS”.

### 2.2. Sample Characterization

The crystallographic properties are studied through the X-Ray Diffraction (XRD) patterns recorded with a Bruker D8 Twin-Twin powder diffractometer using Cu-K_α_ radiation. The Scherrer formula (Equtaion (1)) is used to determine the size of the TiO_2_ crystallites, *d*_XRD_ [35]:

(1) $$d_{\mathrm{XRD}}=0.9\frac{\lambda }{B\cos\left(\theta \right)}$$

where *d*_XRD_ is the crystallite size (nm), *B* the peak full-width at half maximum after correction of the instrumental broadening (rad), *λ* the X-ray wavelength (0.154 nm), and *θ* the Bragg angle (rad). 

The repartition of the crystallographic phases is estimated with the Rietveld method using *“*Profex*”* software (Profex 3.12.1, Nicola Döbelin, Solothurn, Switzerland) [36]. The amount of crystalline phase is estimated with CaF_2_ internal standard (calcium fluoride, Sigma-Aldrich, anhydrous powder, 99.99% trace metal basis), also using *“*Profex*”* software [37].

The TiO_2_ textural properties are characterized by nitrogen adsorption-desorption isotherms in an ASAP 2420 multi-sampler adsorption-desorption volumetric device from Micromeritics. From these isotherms, the microporous volume is calculated from the Dubinin-Radushkevich theory (*V*_DR_). The surface area is evaluated using the Brunauer, Emmett and Teller theory (*S*_BET_) [38]. An average particle size, *d*_BET_, can be calculated from *S*_BET_ values by assuming non-porous TiO_2_ anatase nanoparticles using the following formula [7]:

(2) $$\frac{d_{\mathrm{BET}}}{6}=\frac{\frac{1}{\rho_{\mathrm{anatase}}}}{S_{\mathrm{BET}}}$$

where *ρ*_anatase_ is the apparent density of TiO_2_-anatase considered equal to 3.89 × 10^6^ g·m^−3^ [8,9].

The sizes of TiO_2_ nanoparticles are estimated by transmission electron microscopy (TEM) by averaging the measurement of approximately 100 particles on TEM micrographs obtained with a Phillips CM 100 device (accelerating voltage 200 Kv, Amsterdam, The Netherlands). First, samples are dispersed in distilled water using an ultrasonic treatment. Then a drop of the dispersion is placed on a copper grid (Formvar/Carbon 200 Mesh Cu from Agar Scientific, Essex, UK). 

The sample’s optical properties are evaluated by using diffuse reflectance spectroscopy measurements in the region 300–800 nm with a Varian Cary 500 UV–Vis-NIR spectrophotometer, equipped with an integrating sphere (Varian External DRA-2500, Palo Alto, CA, USA) and using BaSO_4_ as reference. The UV–Vis spectra recorded in diffuse reflectance (*R*_sample_) mode are transformed by using the Kubelka–Munk function:

(3) $$F\left(R_{\infty }\right)=\frac{{\left(1-R_{\infty }\right)}^{2}}{2R_{\infty }}$$

where *R*_∞_ is defined as *R*_∞_ = *R*_sample_/*R*_reference_ [8,39,40] with *R*_reference_, the diffuse reflectance measured for the BaSO_4_ reference. To compare them, all spectra are normalized to 1.0 by dividing each spectrum by its maximum intensity [7,41]. Using the well-known equation:

(4) $${\left(F\left(R_{\infty }\right)h\nu \right)}^{1/m}=C\;\left(h\nu -E_{g}\right)$$

where C is a constant and m is a constant that depends on the optical transition mode, the direct and indirect optical band-gap values, *E*_g,direct_ (eV) and *E*_g,indirect_ (eV) are obtained by plotting, respectively, (*F*(*R*_∞_)h*ν*)^2^ and (*F*(*R*_∞_)h*ν*)^1/2^ as functions of the photon energy h*ν* and by determining the intersection of the linear part of the curve and the *x*-axis [8,42].

X-ray photoelectron spectra are obtained with a SSI-X-probe (SSX-100/206) spectrometer equipped with a monochromatized microfocused Al X-ray source (1486.6 eV), operating at 10 kV and 20 mA. Samples are placed in the analysis chamber, where the residual pressure was of about 10^−6^ Pa. The charging effect is adjusted using flood gun energy at 8 eV and a fine-meshed nickel grid placed 3 mm above the sample surface [43]. The pass energy is 150 eV, and the spot size is 1.4 mm^2^. Angle between the normal to the sample surface and the direction of electron collection is 55°. Under these conditions, the mid-height width (FWHM) of the Au 4*f_7/2_* peak photo-peak measured on a standard sample of cleaned gold is about 1.6 eV. The following sequence of spectra is recorded: general spectrum, C 1*s*, O 1*s*, N 1*s* and Ti 2*p* and again C 1*s* to check the stability of charge compensation with time and absence of degradation of the samples.

The C–(C,H) component of the carbon C 1*s* peak is fixed at 284.8 eV to calibrate the scale in binding energy. Two other components of the carbon peak (C–(O,N), C=O or O–C–O) are resolved, notably to determine the amount of oxygen due to contamination. The O–C=O component that would show up at slightly higher binding energy is not observed in our samples. Data processing is carried out with the CasaXPS program (Casa Software Ltd., Teignmouth, UK). Some spectra are decomposed using the Gaussian and Lorentzian function product model (least squares fitting) after subtraction of a nonlinear baseline [44]. The molar fractions are calculated using the normalized peak areas based on acquisition parameters and sensitivity factors supplied by the manufacturer.

### 2.3. Photocatalytic Tests

The photocatalytic activity of the samples in the form of powders is evaluated by following the degradation of *p*-nitrophenol (PNP) after 0, 8 and 24 h, in triplicate, in water medium. For each test, the degradation percentage of PNP, *D*_PNPi_, is given by Equation (5) [9]:

(5) $$D_{PNPi}\left(\%\right)=\left(1-\frac{{\left[PNP\right]}_{i}}{{\left[PNP\right]}_{0}}\right)\times 100$$

where *[PNP]*_i_ represents the residual concentration of PNP at time *t* = *i* h and *[PNP]*_0_ represents the initial concentration of PNP at time *t* = 0 h.

The photocatalytic activity of the samples is estimated under halogen light (UV/visible light) and under the same lamp covered with a UV-filter that removes wavelengths lower than 390 nm, this condition will be called low-energy light [9]. The experimental setup is shown in [8,45]. The test is the same as the procedure described in [8,9]. The concentration of PNP is estimated by UV/Vis spectroscopy (GENESYS 10S UV–Vis from Thermo Scientific, Waltham, MA, USA) at 318 nm. For each catalyst tested, three flasks are exposed to light to calculate the PNP degradation and one is kept in the dark (dark test) to evaluate PNP adsorption on the samples [8,9]. Additionally, a flask with only PNP without any catalyst is exposed to the light for 24 h (blank test), to show that no natural PNP concentration occurs under halogen illumination. In each flask, the initial concentration of catalyst and PNP are 1 g·L^−1^ and 10^−4^ M, respectively [8,9]; the initial pH is 4. Experiments are conducted in test tubes fitted with a sealing cap. These tubes are placed in a cylindrical glass reactor with the halogen lamp in the center. The halogen lamp has a continuous spectrum from 300 nm (or 390 nm with UV filter) to 800 nm (300 W, 220 V), measured with a Mini-Spectrometer TM-UV/vis C10082MD from Hamamatsu (Hamamatsu, Japan) [8,9]. The reactor is maintained at constant temperature (20 °C) by a cooling system that functions by recirculating water; the lamp is also cooled by a similar system [8,9]. Aluminum foil is used to cover the outer wall of the reactor to prevent any interactions with the room lighting. The volume of each flask is 10 mL, agitated by a magnetic stirrer. The PNP degradation due to photocatalysis is equal to the total PNP degradation minus the PNP adsorption estimated with the dark test [8,9].

## 3. Results

### 3.1. X-ray Diffraction (XRD) of TiO_2_ Samples

**Figure 1:**
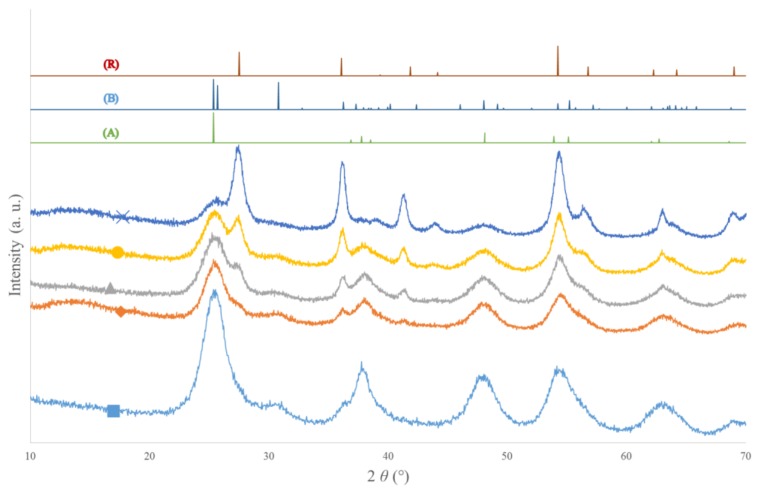
X-ray diffraction (XRD) patterns of ethylenediamine-doped samples: (■) TiO_2_ pure B, (♦) TiO_2_/EtDN1, (▲) TiO_2_/EtDN2, (●) TiO_2_/EtDN4 and (⨯) TiO_2_/EtDN10. (A) reference pattern of anatase, (B) reference pattern of brookite and (R) reference pattern of rutile.

Figure 1 represents the XRD patterns for TiO_2_ pure B sample and ethylenediamine-doped samples. 

For TiO_2_ pure B sample, mainly anatase peaks are observed (reference pattern (A)), along with a peak corresponding to brookite phase at around 31° (reference pattern (B)). The phase distribution calculated with *“*Profex*”* [36] is given in Table 1. Indeed, the anatase phase is the main crystalline phase of this sample, corresponding to 75% of the sample, the brookite phase amounts to around 10%, and the amorphous fraction corresponds to 15%. 

When the amount of ethylenediamine increases, the intensity of rutile peaks increases and that of anatase peaks decreases. This observation is confirmed by the phase distribution in Table 1; for example, for the TiO_2_/EtDN1 sample, the repartition is 65% anatase, 10% rutile and 5% brookite, while for the TiO_2_/EtDN10 sample, the repartition is modified, with 20% anatase, 40% rutile and 5% brookite. The TiO_2_/EtDN10 sample has the biggest amorphous fraction, reaching 35%.

For the TiO_2_ pure A sample, the urea-doped and trimethylamine-doped samples (not shown), the same XRD patterns are observed as for the TiO_2_ pure B sample (mainly anatase + a small fraction of brookite phase). For these samples, the phase distributions are given in Table 1. Similar repartitions are obtained with mainly anatase (65–75%), a small amount of brookite (5–10%) and an amorphous fraction (20–30%).

The crystallite size can be estimated from XRD patterns using the Scherrer formula (Equation (1)). For all samples, similar sizes are obtained between 4 and 8 nm (Table 1). When several phases are present, the size is estimated on specific peaks corresponding to each of the different phases. If different values are obtained, it is mentioned in Table 1 (as for TiO_2_/EtDN4 sample, where anatase crystallites are around 5 nm and rutile around 8 nm). The formation of rutile phase in ethylenediamine-doped samples seems to produce larger crystallites (Table 1).

### 3.2. TEM Micrographs

**Figure 2:**
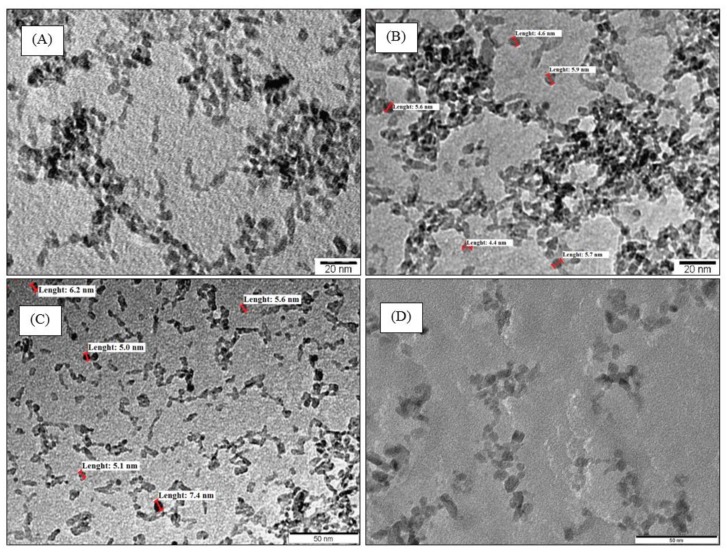
TEM micrographs of samples: (**A**) TiO_2_ pur, (**B**) TiO_2_/U2, (**C**) TiO_2_/EtDN4 and (**D**) TiO_2_/Et_3_N42. Primary nanoparticles are highlighted by red lines.

TEM micrographs for some samples at different magnifications are presented in Figure 2. For all samples, TiO_2_ aggregates are observed; these aggregates are composed of TiO_2_ nanoparticles with spherical shapes. The particles are not perfectly separated from each other because the material was first dried and then deposited on the TEM grid for measurement; additionally, the TiO_2_ nanoparticles are not very clearly observable by TEM because of their relatively low contrast in bright-field TEM conditions [9]. TiO_2_ nanoparticles have a size of about 5–7 nm. The TiO_2_ average size estimated from TEM (*d*_TEM_) is similar to that estimated from XRD (*d*_XRD_, Table 1).

### 3.3. Sample Textural Properties

**Figure 3:**
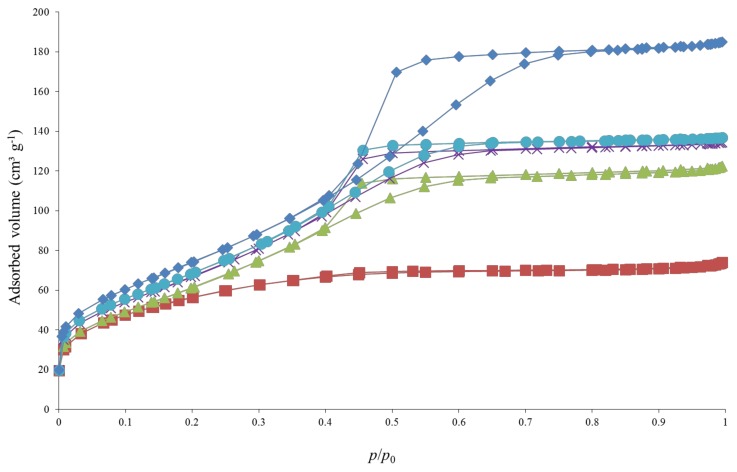
Nitrogen adsorption-desorption isotherms of urea doped samples: (■) TiO_2_ pure A, (▲) TiO_2_/U1, (⨯) TiO_2_/U2, (●) TiO_2_/U4 and (♦) TiO_2_/U10.

**Figure 4:**
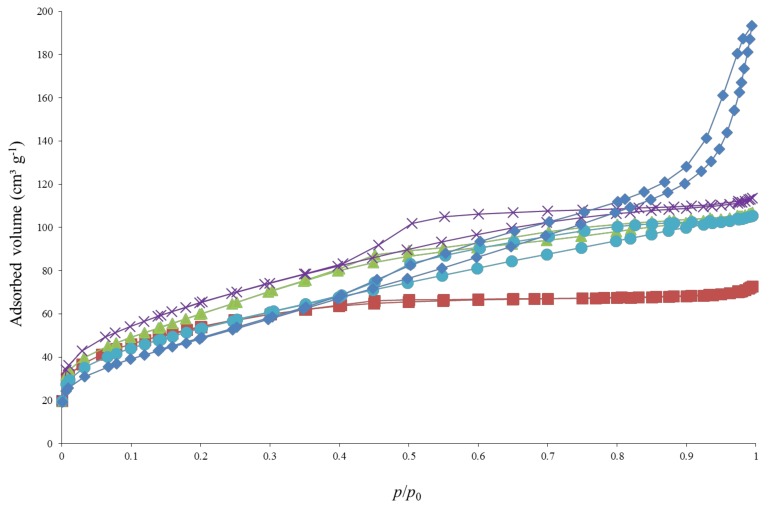
Nitrogen adsorption-desorption isotherms of ethylenediamine doped samples: (■) TiO_2_ pure B, (▲) TiO_2_/EtDN1, (⨯) TiO_2_/EtDN2, (●) TiO_2_/EtDN4 and (♦) TiO_2_/EtDN10.

The textural properties of the samples are summarized in Table 1. Figure 3 and Figure 4 represent the raw nitrogen adsorption-desorption isotherms for the urea-doped and ethylenediamine-doped samples, respectively, with the corresponding pure TiO_2_ sample as reference.

For both pure TiO_2_ samples, the isotherms are characterized by a sharp increase at low relative pressure followed by a plateau, corresponding to type I isotherms (microporous materials) according to the BDDT classification [38].

For the urea-doped samples (Figure 3), the same shape of isotherms is obtained for the 4 samples, presenting a sharp increase at low pressure, and a triangular hysteresis followed by a plateau. This type of isotherm corresponds to a mix of type I (microporous materials) and IV (mesoporous materials) isotherms according to the BDDT classification [38]. The specific surface area, *S*_BET_, and the microporous volume, *V*_DR_, increase with the amount of urea introduced in the synthesis (Table 1).

For the ethylenediamine-doped samples (Figure 4), the same shape of isotherms is obtained for the 3 first samples, presenting a sharp increase at low pressure, and a triangular hysteresis followed by a plateau. This type of isotherm corresponds to a mix of type I (microporous materials) and IV (mesoporous materials) isotherms according to the BDDT classification [38]. The TiO_2_/EtDN10 sample presents a different shape, with a less marked nitrogen uptake at low *p*/*p*_0_ values and a hysteresis extending from *p*/*p*_0_ = 0.5 to *p*/*p*_0_ = 1,which corresponds to larger mesopores [46]. This type of isotherm corresponds to a mix of type I (microporous materials) and IV (mesoporous materials) isotherms according to the BDDT classification [38]. The specific surface area, *S*_BET_, and the microporous volume, *V*_DR_, first increases with the doping ratio then decreases (Table 1).

For the trimethylamine-doped samples, similar isotherms compared to isotherms for the urea-doped samples are obtained (as Figure 3), corresponding to a mix of type I (microporous materials) and IV (mesoporous materials) isotherms according to the BDDT classification [38]. The specific surface area, *S*_BET_, and the microporous volume, *V*_DR_, increase with the amount of triethylamine (Table 1).

For all samples, an estimation of TiO_2_ nanoparticles sizes (*d*_BET_) can be calculated from Equation (2), and in all cases, the TiO_2_ sizes are between 6–8 nm (Table 1), corresponding to both the TEM and XRD estimates.

### 3.4. Optical Properties

**Figure 5:**
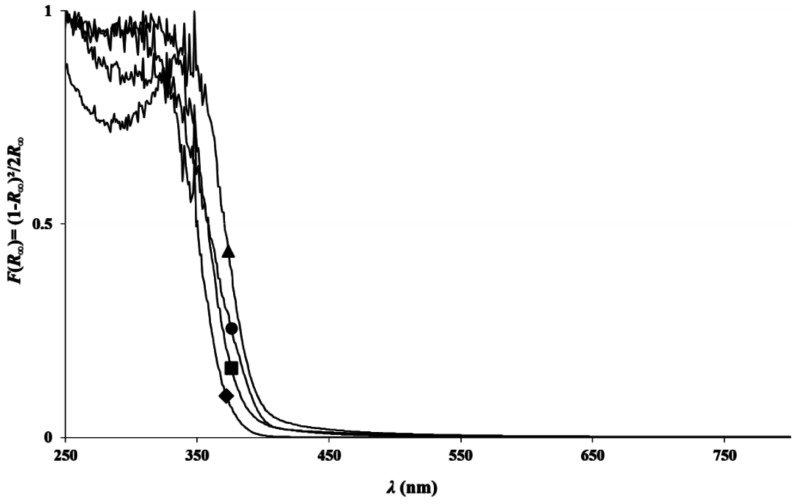
Normalized Kubelka–Munk function F(R_∞_) calculated from DR-UV–Vis spectra for samples: (■) TiO_2_ pure A, (▲)TiO_2_/U2 samples, (♦) TiO_2_ pure B and (●)TiO_2_/EtDN4 samples.

The evolution of the normalized Kubelka–Munk function F(*R*_∞_) with wavelength (*λ*) is presented for TiO_2_ pure A, TiO_2_/U2, TiO_2_ pure B and TiO_2_/EtDN4 samples in Figure 5. The TiO_2_ pure A and B samples present absorption at 360 nm, while for both N-doped samples, the absorption is shifted towards higher wavelengths. Both samples present the largest shift in their respective series. For the other doped samples, although smaller, shifts towards the visible domain are also observed. The direct and indirect band gaps are calculated for all samples (Table 1); a decrease in the band gap value is observed for the N-doped samples compared to the corresponding pure TiO_2_ samples. For the triethylamine doped samples, the absorption spectrum is not exploitable because of the dark color of the sample; therefore, the band gap values cannot be properly calculated.

### 3.5. XPS Measurements

**Figure 6:**
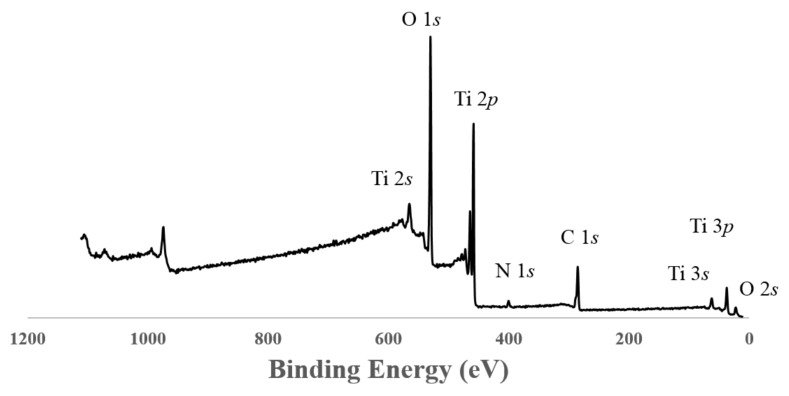
XPS general spectrum of TiO_2_ pure B sample.

**Figure 7:**
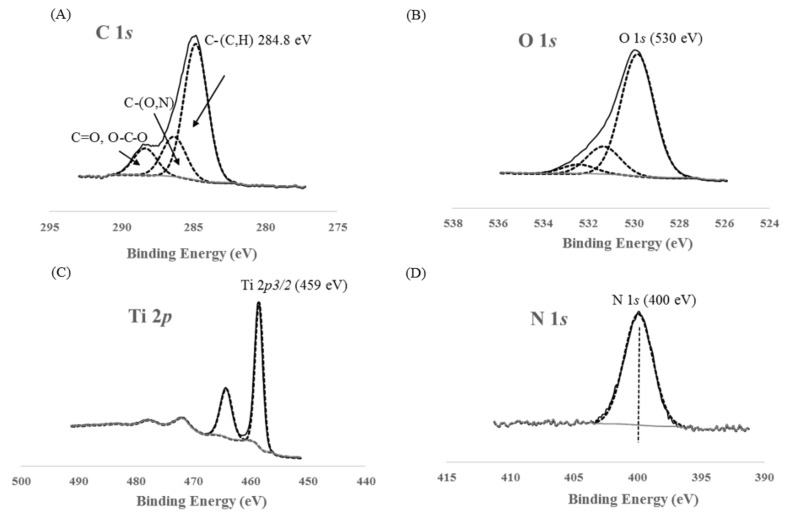
XPS spectra of TiO_2_ pure B sample: (A) C 1s region, (**B**) O 1s region, (**C**) Ti 2p region and (**D**) N 1s region.

The general XPS spectrum for the TiO_2_ pure B sample is presented in Figure 6. The different peaks for carbon, oxygen and titanium are indexed. It is important to note that nitrogen is present in the TiO_2_ reference sample. N 1*s*, Ti 2*p*, O 1*s* and C 1*s* spectra are presented on Figure 7. For all samples, similar spectra are obtained. On the Ti 2*p* spectrum (Figure 7c), the Ti 2*p*1/2 and Ti 2*p*3/2 are observed at 464.1 and 458.5 eV, respectively, and are attributed to Ti^4+^ species, and thus to TiO_2_. No differences between samples are noticeable in the Ti 2*p* area. Indeed, the shift to a slightly lower binding energy induced by a change in the titanium chemical environment (as in the case of Ti–O–N bonding) would be too small to be distinguished from the main peak [47]. On the other hand, the systematic absence of a peak around 455–456 eV excludes the presence of Ti–N species. 

The C 1*s* contribution is divided into three components (Figure 7a). The C–(C,H) contribution at 248.8 eV is a classical aliphatic carbon contamination used to calibrate the measurements. In our standard routine procedure for decomposing the C 1*s* contribution, we define the contribution of carbon involved in simple bond with O or N at 1.5 eV higher (286.3 eV). The signal at a binding energy of around 289 eV is attributed to the contribution of C doubly bonded to O. The pure TiO_2_ samples contain 17 to 19% (C–H) component. The other samples show comparable decomposition of the C signal. This suggests that there are no remaining organic compounds present.

The O 1*s* contribution (Figure 7b) is decomposed into 3 peaks. The main one at 530 eV corresponds to Ti–O in TiO_2_. For each sample, the O 1*s* (530 eV)/Ti *2p* ratio is close to 2, corresponding to stoichiometric TiO_2_. The two other components at higher binding energy are complicated to attribute, as they correspond to oxygen bonded to carbon due to carbon contamination and/or nitrogen bonding (N–O–Ti, N=O) [47].

**Figure 8:**
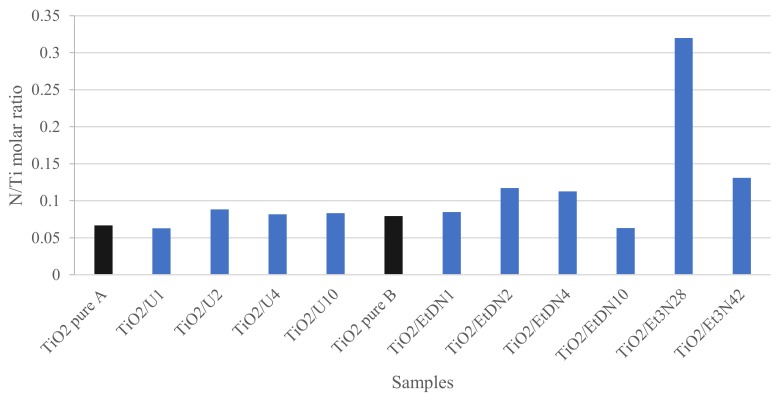
Estimated N/Ti molar ratio calculated from XPS measurements.

**Figure 9:**
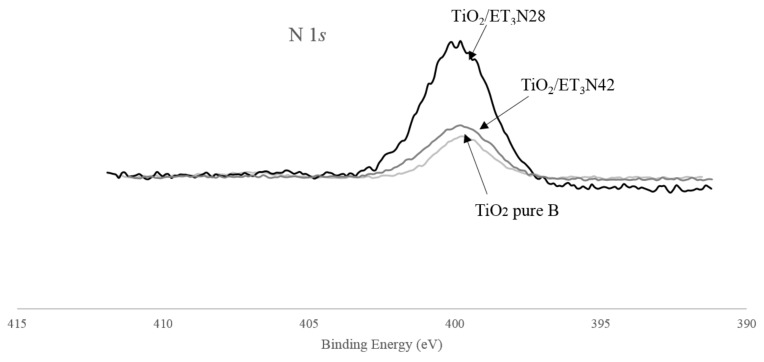
Comparison of normalized N 1*s* region XPS spectra of TiO_2_ pure B, TiO_2_/Et_3_N28 and TiO_2_/Et3N42 samples.

For the N 1*s* spectrum (Figure 7d), a peak centered on 400 eV is observed. For 3 samples (TiO_2_ pure A, TiO_2_/EtDN2 and TiO_2_/EtDN4), a small peak around 407 eV was also observed. This peak has been attributed to residual nitrate due to the residual nitric acid from the synthesis. This contribution was not taken into account in the latter quantification. In the literature, a N 1*s* peak around 400 eV may correspond to many contributions such as NH_x_, NO_x_, NHOH potentially due to impurities but also interstitial Ti–O–N [47,48]. Substitutional Ti–N–O can be excluded in our case, since the specific corresponding peak around 395 eV is never observed. At this stage, the complexity of the decomposition of the N 1*s* peak does not allow to claim the presence of Ti–O–N species only. An estimation of the amount of nitrogen present in the samples (N/Ti molar ratio) is presented in Figure 8 and Table 2 for each series in front of the corresponding pure TiO_2_ sample. An increase in the amount of nitrogen is observed with the three precursors. For each series, a sample presents a maximum in nitrogen concentration: TiO_2_/U2, TiO_2_/EtDN2 and TiO_2_/Et_3_N28 show the three maxima for the urea, the ethylenediamine and the triethylamine doping, respectively. The highest nitrogen amount is obtained with TiO_2_/Et_3_N28 sample with a N/Ti molar ratio of 0.32. The results are presented in Figure 8 and Table 2, and are illustrated in Figure 9 for the triethylamine series.

### 3.6. Photocatalytic Activity

#### 3.6.1. Under UV/Visible Light

For each sample, the test in the dark shows no PNP adsorption on the catalyst. Moreover, no spontaneous PNP degradation appears under UV/visible light in the absence of the catalyst. The catalytic test is performed over 24 h with an estimation of PNP degradation (*D*_PNPi_,%) after *i* = 0, 8 and 24 h. *D*_PNP8_ is used to compare the different catalytic activities of the samples because the differences between catalysts are the most noticeable after 8 hours of activitiy [9].

**Figure 10:**
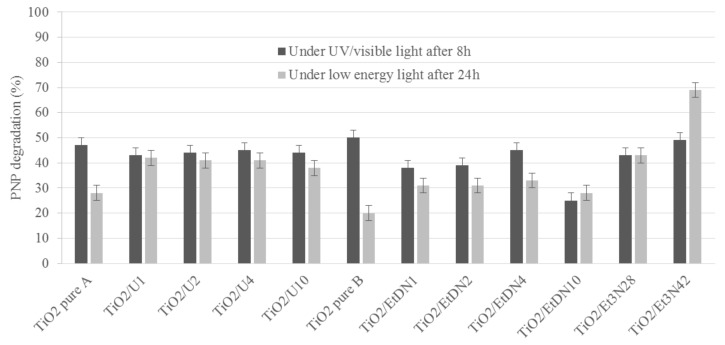
PNP degradation (%) for all samples under UV/visible light after 8 h of irradiation (dark grey) and under low-energy light (with filter to remove λ lower than 390 nm) after 24 h of irradiation (light grey).

*D*_PNP8_ is presented in Figure 10 (dark grey) and in Table 2 for all samples. For both TiO_2_ pure A and B samples, the same activity is obtained at around 50%. Concerning the urea- and trimethylamine-doped samples, the photoactivity stays nearly constant for each sample at around 45–50% of PNP degradation. For the ethylenediamine-doped samples, the activity decreases for all samples when the amount of ethylenediamine increases except for the TiO_2_/EtDN4 sample, which presents 45% of PNP degradation.

**Figure 11:**
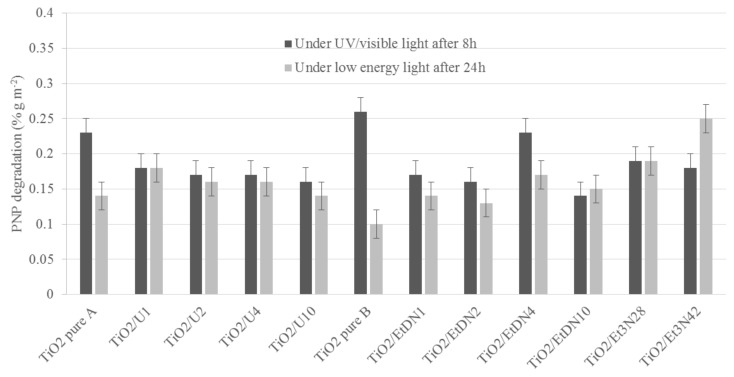
PNP degradation (% g·m^−2^) calculated by dividing the PNP degradation in% by S_BET_ for all the samples under UV/visible after 8 h of irradiation (dark grey) and under low-energy light (with filter to remove λ lower than 390 nm) after 24 h of irradiation (light grey).

If the photocatalytic activity is divided by the specific surface area, *S*_BET_, the same observations can be made (Figure 11 in dark grey).

#### 3.6.2. Under Low-Energy Light (λ > 390 nm)

For low-energy light conditions, *D*_PNP24_ is used to compare the different catalytic activities of samples because under visible light, the reactions are slower [9].

*D*_PNP24_ is presented in Figure 10 (light grey) and Table 2 for all samples. For TiO_2_ pure A and B samples, the activity is slightly different between both syntheses with values of 28 and 20% for the PNP degradation respectively. For the urea-doped samples, the photoactivity increases compared to the corresponding TiO_2_ pure A sample, exhibiting between 39% and 42% PNP degradation. For the ethylenediamine-doped samples, the photoactivity also increases compared to the corresponding TiO_2_ pure B sample, with the optimal value being obtained for the TiO_2_/EtDN4 sample, with 33% PNP degradation. For the trimethylamine-doped samples, the photoactivity also increases compared to the corresponding TiO_2_ pure B sample with the optimal value obtained for the TiO_2_/Et_3_N42 sample, with 69% PNP degradation.

If the activity is divided by the specific surface area, *S*_BET_, the same observations can be made (Figure 11, in light grey).

Concerning the use of UV/visible spectroscopy to measure the PNP degradation, it has been reported in the literature [49,50] that the presence of intermediate species associated with the partial degradation of PNP can be detected by the presence of peaks corresponding to the intermediates (4-nitrocatechol, 1,2,4-benzenetriol, hydroquinone) in the UV/Vis spectrum measured between 200 and 500 nm after several hours under illumination [9]. In the present study, no supplementary peaks are measured in the UV/Vis spectra between 200 and 500 nm, which is consistent with the complete mineralization of the pollutant, and therefore it is concluded that the photocatalysts developed in this study promote the complete mineralization of PNP [9]. Furthermore, total mineralization of PNP during homologous photocatalytic tests on a similar installation has been shown in a previous work [9,10].

### 3.7. Characterization of the Large-Scale Urea-Doped TiO_2_ Photocatalyst 

A urea-doped TiO_2_ sample (TiO_2_/U2-LS) is successfully synthesized at a large scale (5 L). It is characterized by XRD and BET measurements, and its photocatalytic activity is tested. All the results are shown in Table 1 and Table 2. Concerning the physico-chemical properties, analogous characteristics are obtained compared to the laboratory scale sample (TiO_2_/U2), with mainly anatase TiO_2_ nanoparticles with a size of 7 nm and an *S*_BET_ value of 245 m^2^·g^−1^ (Table 1). For the photocatalytic activity, similar values are obtained as for the laboratory scale sample under UV/visible and low-energy light, namely 46% and 43%, respectively (Table 2).

## 4. Discussion

All these syntheses are based on the precipitation-acidic peptization of a titanium precursor, where the acid allows dissolving amorphous TiO_2_ precipitate by hydrolysis followed by the crystallization of small TiO_2_ nanoparticles. This leads to the formation of stable crystalline TiO_2_ colloids [51].

### 4.1. Crystallinity and Texture of TiO_2_ Based Samples

Urea and triethylamine doping does not influence the crystallinity of the samples. Indeed, the TiO_2_ phase distribution stays nearly constant between the pure and doped samples (Table 1). For these dopings, the synthesis conditions have not been modified and so same crystalline phases are obtained (anatase and brookite, Table 1). On the opposite, ethylenediamine doping modifies clearly the TiO_2_ phase distribution leading to the formation of rutile TiO_2_ when the amount of dopant increases (Table 1). For these syntheses, the amount of nitric acid has to be increased to obtain a peptization process of titania precursor. Indeed, ethylenediamine has a strong basic character [52], which requires more acid to reach peptization. In this case, if the amount of acid is increased, more oxolation bonds between titanium atoms are broken and more OH groups are produced around a single titanium atom [53], which facilitates the crystallization of TiO_2_ in its most thermodynamically stable phase, i.e., the rutile phase [51,53].

Concerning the shape of the samples, TEM pictures, BET measurements and XRD crystallite size (Table 1), similar values between 4 and 8 nm are obtained. It is assumed that the samples are made of TiO_2_ nanoparticles where a nanoparticle corresponds to a crystallite [7,54].

For the influence of doping on the texture, a modification of the isotherms compared to pure TiO_2_ samples is observed. Indeed, doping leads to the formation of mesopores. For urea and triethylamine doping, the addition of dopant modifies the electrostatic interactions between the TiO_2_ particles due to the incorporation of nitrogen, leading to a different particle stacking compared to pure TiO_2_ samples when the samples are dried [54]. The difference of stacking between pure and doped TiO_2_ nanoparticles has been previously described in [54]; it becomes less compact with doping which creates the mesoporosity [54]. Concerning ethylenediamine doping, two effects modify the isotherms: (i) the modification of the electrostatic interactions between the TiO_2_ particles due to the incorporation of nitrogen as for the other dopings, and (ii) the modification of the crystalline phase distribution. In this second case, the apparition of rutile induces a change in the shape of the N_2_ physisorption isotherm as described in [18]. Indeed, when the amount of ethylenediamine increases, mesopores appear (hysteresis of TiO_2_/EtDN10 sample on Figure 4) due to the effect of N-doping and the amount of rutile remains <20%. However, when the amount of rutile exceeds 50%, the hysteresis is modified and reveals larger pores due to the rutile [18], which seems to produce larger rutile crystallite of 8 nm compared to anatase crystallite of 5 nm (*d*_XRD_ in Table 1). Concerning the two pure TiO_2_ syntheses, these lead to similar materials as their specific surface area, crystalline phase and particle shapes are analogous (Table 1).

### 4.2. Photoactivity and N-Doping

For pure TiO_2_ samples, the degradation of PNP (*D*_PNPi_) is 47 and 50% after 8 h under UV/visible light for TiO_2_ pure A and B, respectively, while it is 28 and 20% after 24 h under low-energy light. The activity under low-energy light is the characteristic of interest in this research, as an efficient visible photocatalyst is aimed for. This low-energy light is mainly “visible light”, as the UV filter removes λ < 390 nm.

If both syntheses are compared, similar activities are obtained, which is consistent with the same type of materials that are observed through the characterizations (Table 1). An activity under low-energy light is observed for both catalysts. At the same time, XPS measurements (Figure 7d and Figure 8) show that nitrogen is still present in both pure materials, with the peak at 400 eV seeming to correspond to Ti–O–N bonds [55,56]. So, a small nitrogen doping has occurred due to nitric acid used during the synthesis. A comparison is made with the commercial Degussa P25 catalyst, which has no trace of nitrogen [57] and is poorly active under visible light [57,58]. In this case, the PNP degradation after 24 h is merely 10%, which could be induced by the remaining UV radiation, which is not removed by the UV filter as explained above. Therefore, the TiO_2_ pure A and B samples have an activity under low energy (and better than P25) not only due to the remaining UV light, but likely due to a trace of N-doping.

Concerning the N-doped samples, an increase in the activity is observed for all samples compared to the corresponding pure TiO_2_ materials. All samples present a peak at 400 eV, which may correspond to Ti–O–N bonds [47,48,55,56].

**Figure 12:**
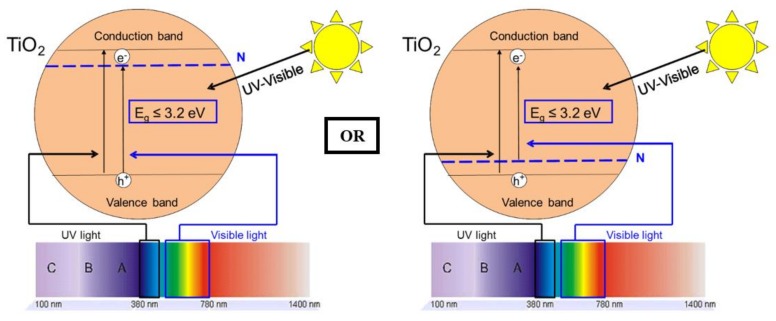
Effect of N-doping on TiO_2_ photocatalysis.

For the urea-doped samples, XPS measurements (Figure 8) and diffuse reflectance spectroscopy (Figure 5) show an N-doping higher than the corresponding TiO_2_ pure A sample and a reduction of the band gap value (Table 1). In this case, the nitrogen doping has been effective. As explained in [30,31,33], nitrogen doping can reduce the band gap by creating an intermediate band for the electron below the conduction band or above the valence band (Figure 12). It seems that in the four samples, the amount of nitrogen introduced in the samples is quite similar, even if different amounts were added during the synthesis, leading to materials with similar photocatalytic activity (*D*_PNP24_ between 37 and 42%) and optical properties (similar band gaps, Table 1). Urea seems to be a N precursor effective for doping, leading to visible active photocatalyst as described in [23].

Concerning the ethylenediamine-doped samples, the doping seems to also produce effective visible photocatalysts. Indeed, the activity increases for the four samples (Table 2), along with the band gap decreases (Table 1), and the amount of nitrogen increases for the TiO_2_/EtDN2 and TiO_2_/EtDN4 samples (Figure 8). The activity under low-energy light evolves, depending on the amount of nitrogen; when the amount increases, the activity increases (from samples TiO_2_/EtDN1 to TiO_2_/EtDN4, see Table 2 and Figure 8), but for the TiO_2_/EtDN10 sample when the amount decreases, the activity decreases. The bad gap values follow the same trend (Table 1). Another important aspect for this series is that rutile TiO_2_ is present in different percentages for the different samples, which can impact the photocatalytic activity. The rutile phase increases from TiO_2_/EtDN1 sample to TiO_2_/EtDN10 sample, but rutile is a less active phase than the anatase phase [6] and is also poorly active under visible light [6,24]. Therefore, the activity would decrease through the samples, but it is not observed under UV/visible light. Hence, the N-doping is successful and clearly linked to the activity of samples. On the other hand, a synergetic effect between anatase and rutile can also exist [24], leading to a better activity for the TiO_2_/EtDN4 sample with the composition of 45% anatase and 35% rutile. This synergetic effect is also observed for Degussa P25, which has a composition of 80% anatase and 20% rutile [59]. A same mechanism for nitrogen doping as for urea doping can thus be considered (Figure 12).

For the trimethylamine-doped samples, the best activity is observed especially for the TiO_2_/Et_3_N42 sample, which reaches 69% PNP degradation. For this series of samples, the band gap values are not available as explained in Section 3.4. Only XPS measurements can therefore be used and show an increased nitrogen amount compared to the TiO_2_ pure B sample (Figure 8), and that the N amount is the highest, when compared to the other series of samples. The very good activity shows that the N-doping is effective as described in [23,55] and a same mechanism for nitrogen doping as for urea and ethylenediamine doping can thus be considered (Figure 12).

The three precursors show that visible N-doped catalyst is obtained at low temperature, and that triethylamine seems to be the best nitrogenous agent, but requires the largest amount of precursors for doping.

### 4.3. Large-Scale Synthesis

For a visible light application, large amounts of catalyst will be needed, this is why a large-scale synthesis of N-doped TiO_2_ of 5 L has been performed. The urea precursor is chosen because of its easy availability, low cost and low toxicity [60]. The urea/Ti precursor ratio of 2 has been chosen according the good results obtained for the TiO_2_/U2 sample at laboratory scale, and is thereby denoted TiO_2_/U2-LS. 

The results show that the large-scale synthesis does not influence the physico-chemical and photocatalytic properties of urea-doped TiO_2_ samples (Table 1 and Table 2). The PNP degradation of this large-scale sample is also higher than P25 activity under low-energy light (Table 2). 

If this sample is compared to the commercial catalyst Degussa P25, the main advantages are the following [9]: (i) crystalline urea-doped TiO_2_ samples are obtained without a calcination step; (ii) the synthesis is better for the environment, as water is the solvent (low use of organic compounds); (iii) the synthesis protocol is easy; (iv) the only cost is the TiO_2_ precursor (titanium tetraisopropoxide) and urea, while the synthesis of commercial Degussa P25 involves the use of an aerosol process, which is known to be expensive, environmentally “unfriendly”, and involves high-temperature treatments (1000–1300 °C) [7,61]; and (v) the sample is 4 times more active than P25 under visible light.

## 5. Conclusions

In this chapter, the previously developed aqueous titania sol-gel synthesis is doped with nitrogen precursors to extend its activity towards visible region. Three N-precursors are used: urea, ethylenediamine and triethylamine. Different molar ratios have been tested for each dopant. Results showed the formation of anatase-brookite TiO_2_ nanoparticles of 6–8 nm with a specific surface area of between 200 and 275 m^2^·g^−1^ for the urea and triethylamine series. Using ethylenediamine, the formation of rutile phase is observed when the amount of ethylenediamine increases due to the addition of nitric acid in order to maintain the peptization process. In this series, TiO_2_ nanoparticles of 6–8 nm are also obtained with a specific surface area between 185 and 240 m^2^·g^−1^.

The combination of XPS and diffuse reflectance measurements suggests the incorporation of nitrogen in the TiO_2_ materials through Ti–O–N bonds, allowing absorption in the visible region. Catalytic tests show a marked improvement of performance under visible radiation for all doped catalysts in the remediation of polluted water with *p*-nitrophenol. In this case, nitrogen doping can reduce the band gap by creating an intermediate band for the electrons below the conduction band or above the valence band, allowing activity in the visible range. 

The best doping, taking into account cost, activity and ease of synthesis, is up-scaled to a volume of 5 L and compared to the commercial Degussa P25 material. This urea-doped large-scale catalyst shows analogous properties as the lab-scale corresponding synthesis and photoactivity 4 times higher than the commercial catalysts Degussa P25.

## Figures and Tables

**Table 1:** Textural and optical properties of TiO_2_-based photocatalysts.

Sample	Phase Distribution	*d*_XRD_	*S*_BET_	*V*_DR_	*d*_BET_	*d*_TEM_	*E*_g,direct_	*E*_g,indirect_
	(%)	(nm)	(m^2^g^−1^)	(cm^3^g^−1^)	(nm)	(nm)	(eV)	(eV)
	±5	±1	±5	±0.01	±1	±1	±0.01	±0.01
TiO_2_ pure A	Am[25%] + A[65%] + B[10%]	4	205	0.11	8	5	3.35	3.06
TiO_2_/U1	Am[30%] + A[65% ]+ B[5%]	5	235	0.15	7	6	3.25	2.97
TiO_2_/U2	Am[30%] + A[65%] + B[5%]	4	255	0.16	6	6	3.24	2.97
TiO_2_/U4	Am[30%] + A[65%] + B[5%]	4	260	0.16	6	7	3.27	3.04
TiO_2_/U10	Am[25%] + A[70%] + B[5%]	4	270	0.16	6	5	3.36	3.05
TiO_2_ pure B	Am[15%] + A[75%] + B[10%]	4	195	0.10	8	5	3.42	3.12
TiO_2_/EtDN1	Am[20%] + A[65%] + B[5%] + R[10%]	7	225	0.13	7	6	3.30	2.94
TiO_2_/EtDN2	Am[15%] + A[60%] + B[5%] + R[20%]	4	240	0.13	6	6	3.39	2.99
TiO_2_/EtDN4	Am[15%] + A[45%] + B[5%] + R[35%]	5 ^a^–8 ^b^	195	0.11	8	7	3.30	2.94
TiO_2_/EtDN10	Am[35%] + A[20%] + B[5%] + R[40%]	8	185	0.11	8	5	3.43	2.96
TiO_2_/Et_3_N28	Am[25%] + A[70%] + B[5%]	4	230	0.13	7	5	-^c^	-^c^
TiO_2_/Et_3_N42	Am[20%] + A[75%] + B[5%]	4	275	0.16	6	6	-^c^	-^c^
TiO_2_/U2-LS	Am[30%] + A[65%] + B[5%]	6	245	0.15	6	7	-^d^	-^d^

Am: amorphous TiO_2_ phase; A: anatase TiO_2_ phase; B: Brookite TiO_2_ phase; R: rutile TiO_2_ phase; *d*_XRD_: mean diameter of TiO_2_ crystallites measured by the Scherrer method; ^a^ measured from anatase peak; ^b^ measured from rutile peak; *S*_BET_: specific surface area determined by the BET method; *V*_DR_: specific micropore volume determined by Dubinin–Raduskevitch theory; *d*_BET_: mean diameter of TiO_2_ nanoparticles calculated from *S*_BET_ values; *d*_TEM_: mean diameter of TiO_2_ nanoparticles measured by TEM; *E*_g,direct_: direct optical band-gap values calculated using the transformed Kubelka–Munk function; *E*_g,indirect_: indirect optical band-gap values calculated using the transformed Kubelka–Munk function; -^c^ not applicable; -^d^ not measured.

**Table 2:** Photocatalytic properties and XPS results of TiO_2_-based samples.

Sample	*D*_PNP8_ under UV/Visible	*D*_PNP8_ under UV/Visible	*D*_PNP24_ under Visible	*D*_PNP24_ under Visible	N/Ti
	(%)	(% g·m^−2^)	(%)	(% g·m^−2^)	(mol/mol)
	±3	±0.02	±3	±0.02	
TiO_2_ pure A	47	0.23	28	0.14	0.07
TiO_2_/U1	43	0.18	42	0.18	0.06
TiO_2_/U2	44	0.17	41	0.16	0.09
TiO_2_/U4	45	0.17	41	0.16	0.08
TiO_2_/U10	44	0.16	38	0.14	0.08
TiO_2_ pure B	50	0.26	20	0.10	0.08
TiO_2_/EtDN1	38	0.17	31	0.14	0.08
TiO_2_/EtDN2	39	0.16	31	0.13	0.12
TiO_2_/EtDN4	45	0.23	33	0.17	0.11
TiO_2_/EtDN10	25	0.14	28	0.15	0.06
TiO_2_/Et_3_N28	43	0.19	43	0.19	0.32
TiO_2_/Et_3_N42	49	0.18	69	0.25	0.13
TiO_2_/U2-LS	46	0.19	43	0.18	-^a^

*D*_PNP8_: the degradation percentage of PNP after 8 h of illumination; *D*_PNP24_: the degradation percentage of PNP after 24 h of illumination, values in% g·m^−2^ are obtained by dividing values in% by S_BET_; N/Ti: molar ration between N and Ti measured by XPS; -^a^ not measured.
